# Alphatoxin Nanopore Detection of Aflatoxin, Ochratoxin and Fumonisin in Aqueous Solution

**DOI:** 10.3390/toxins15030183

**Published:** 2023-02-28

**Authors:** Artur Alves Rodrigues da Silva, Janilson José da Silva Júnior, Maria Isabel dos Santos Cavalcanti, Dijanah Cota Machado, Paloma Lys Medeiros, Claudio Gabriel Rodrigues

**Affiliations:** 1 Education and Health Center, Federal University of Campina Grande, Rua Aprígio Veloso, 882, Universitário, Campina Grande 58429-900, Brazil; 2 Postgraduate Program in Therapeutic Innovation, Federal University of Pernambuco, Avenida Professor Moraes Rego, s/n, Cidade Universitária, Recife 50670-901, Brazil; 3 Department of Biophysics and Radiobiology, Federal University of Pernambuco, Avenida Professor Moraes Rego, s/n, Cidade Universitária, Recife 50670-901, Brazil

**Keywords:** alphatoxin, aflatoxin, stochastic sensing, ochratoxin, fumonisin, biosensor, nanopore

## Abstract

Mycotoxins are toxic and carcinogenic metabolites produced by groups of filamentous fungi that colonize food crops. Aflatoxin B_1_ (AFB_1_), ochratoxin A (OTA) and fumonisin B_1_ (FB_1_) are among the most relevant agricultural mycotoxins, as they can induce various toxic processes in humans and animals. To detect AFB_1_, OTA and FB_1_ in the most varied matrices, chromatographic and immunological methods are primarily used; however, these techniques are time-consuming and expensive. In this study, we demonstrate that unitary alphatoxin nanopore can be used to detect and differentiate these mycotoxins in aqueous solution. The presence of AFB_1_, OTA or FB_1_ inside the nanopore induces reversible blockage of the ionic current flowing through the nanopore, with distinct characteristics of blockage that are unique to each of the three toxins. The process of discrimination is based on the residual current ratio calculation and analysis of the residence time of each mycotoxin inside the unitary nanopore. Using a single alphatoxin nanopore, the mycotoxins could be detected at the nanomolar level, indicating that alphatoxin nanopore is a promising molecular tool for discriminatory analysis of mycotoxins in aqueous solution.

## 1. Introduction

The growth of filamentous fungal species in varied foods and animal feeds results in the production of metabolic substances that, when ingested, can be toxic to humans and animals. These substances are termed mycotoxins, and the toxification process is referred to as mycotoxicosis [1,2,3]. Certain environmental conditions, such as regarding temperature and humidity, are favorable to fungal growth; there is a higher incidence of fungal growth in countries with tropical climates. In particular, species of the genera *Aspergillus*, *Penicillium* and *Fusarium* produce some of the most studied mycotoxins groups, namely, aflatoxins, ochratoxins and fumonisins [1,2,3,4,5,6,7]. Aflatoxin B_1_ (AFB_1_) is the most studied due to its high toxicity and wide presence in food; according to the International Cancer Research Agency, this toxin is a group 1 carcinogenic substance [8,9]. This makes AFB_1_ a significant object of scientific interest in the identification of toxicological pathways for humans and the development of new analytical tools to detect its presence in food crops [10]. Ochratoxins are metabolic products of *Aspergillus* species and some strains of *Penicillium*, and ochratoxin A (OTA) is prevalent in foods [7,11]. Among the homologs of more than 15 fumonisins that are already known, fumonisin B_1_ (FB_1_) is the most abundant and represents the greatest health danger [12]. Brazil is one of the largest grain producers in the world, with studies reporting the presence of these three mycotoxins in many economically important food crops. In particular, studies regarding OTA and FB_1_ report the contamination of two of Brazil’s main exports: coffee and corn [13,14,15]. The risks of exposure to these classes of toxins are due to difficulty controlling their growth in grains as a result of the natural growth of fungi under certain conditions of humidity and temperature during the pre-harvest phase in addition to the toxins’ post-harvest stability and resilience against chemicals, thermal factors and physical treatments [16,17]. Several regulatory bodies, such as the United States Food and Drug Administration (FDA), the World Health Organization (WHO), the Food Agriculture Organization (FAO) and the European Food Safety Authority (EFSA), set strict regulatory guidelines for major mycotoxins in food and animal feed; such guidelines are currently applied by approximately 100 countries [18]. The objective of these regulations is to ensure food safety by avoiding the possible carcinogenic effects indicated by studies that result from the chronic consumption of foods with a high mycotoxin content in addition to surges of food poisoning, with such consumption having been previously identified as the cause of death of hundreds of people in Kenya [19,20,21,22,23]. For the detection and identification of mycotoxins in food and feed, sampling is initially performed, followed by extraction, cleaning and the application techniques that facilitate the detection and quantification of these toxins [24,25]. The methods currently applied in laboratory routines have been validated for this process and include high performance liquid chromatography with fluorimetric detection (HPLC-FLD) [26], liquid chromatography coupled with mass spectrometry (HPLC-MS) [27,28], matrix-assisted laser mass ionization/desorption spectrometry (MALDI-TOF) [29] and enzyme-linked immunosorbent assay (ELISA) [30,31,32]. It is important to emphasize that these conventional methods have a competent detection range and in many cases are multianalytical [29,31]. Chromatography techniques are versatile because they can be used with a variety of detectors. In this way, most matrices, such as corn, coffee, milk and others, can be analyzed to efficiently and sensitively determine the mycotoxin content in these foods in the nano- to picomolar range [24,25,26,27,28,29]. ELISA-based immunoassays are efficiently used, commercial kits for mycotoxins are available and they are simpler techniques; however, they still suffer from incubation steps and cross-reactions [30,31,32]. Such chromatographic and immunological methods are primarily used to detect AFB_1_, OTA, and FB_1_, but are time-consuming and expensive.

Therefore, several studies have sought new methodologies to overcome the drawbacks of the techniques currently applied in identification of these toxins in foods [33,34]. In this context, stochastic biosensors, such as the single alphatoxin protein nanopore, represent a sensitive and low-cost alternative, with the molecular recognition capacity to detect, identify, characterize and quantify single analytes [35]. Stochastic biosensing relies on the observation of individual interactions events between analyte molecules and a single bioreceptor. The interaction between the analyte and the unitary nanopore induces blockage events in the ionic current. The time series formed by these blockage events generates a digital signature that allows the identification and quantification of the analyte [36].

Alphatoxin is an exotoxin with molecular mass of 33.2 kDa produced by *Staphylococcus aureus*, and it forms a heptameric pore that is currently considered the model protein nanopore for the detection of analytes in aqueous solution due to its functional versatility, reproducibility and sensitivity [37,38]. Unitary alphatoxin nanopore is used as a sensing tool in the study of analytes such as nucleotides [39,40,41], polymers [42], peptides and small organic molecules [43,44,45,46,47]. Recently, its advantages in the detection and differentiation of the microcystins, a class of toxins produced by marine algae, were demonstrated [48]. It was also recently demonstrated that aptamers can be used to increase unitary alphatoxin nanopore sensitivity, allowing ultra-sensitive detection of ochratoxin A in corn samples [49]. However, using aptamers increases the cost and complexity of the detection system, limiting its use to the laboratory environment. Another strategy used to improve the detection capability of nanopore-based systems is to increase the salt gradient. Increasing the saline gradient induces electrostatic focusing resulting in an increase in the capture rate of the deoxyribonucleic acid and peptide capture [50,51]. In this study, we demonstrate that single alphatoxin nanopore can be used for the detection and differentiation of the mycotoxins ochratoxin A, aflatoxin B_1_ and fumonisin B_1_ in a solution with of high potassium chloride concentration and absence of a saline gradient.

## 2. Results and Discussion

### 2.1. Mycotoxins Induce Reversible and Characteristic Blockage of Ionic Current in Single Alphatoxin Nanopore

Previous studies denote the ability of nanopores to detect toxins in aqueous solutions. For example, the conductance fluctuation analysis and determination of the all-points histograms allowed the detection and differentiation of microcystin variants by the single alphatoxin nanopore [48]. Here, we performed a residual current and dwell time analysis and demonstrated that the unitary alphatoxin nanopore is able to detect and discriminate three mycotoxins in solution with high KCl concentration.

**Figure 1:**
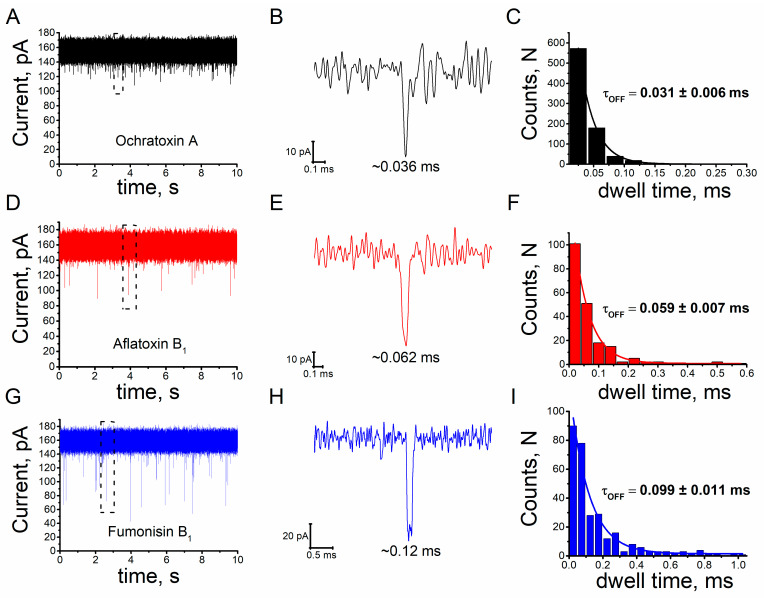
Unitary alphatoxin nanopore detection of mycotoxins. Representative single nanopore ionic current trace for interaction of the alphatoxin nanopore with the mycotoxin (**A**) ochratoxin A, (**D**) aflatoxin B_1_ and (**G**) fumonisin B_1_. Black dashed rectangles indicate blockage events. Representative ionic current blockage events for interaction of the single alphatoxin nanopore with the mycotoxin (**B**) ochratoxin A, (**E**) aflatoxin B_1_ and (**H**) fumonisin B_1_. Representative dwell time histograms for interaction of the single alphatoxin nanopore with the mycotoxin (**C**) ochratoxin A, (**F**) aflatoxin B_1_ and (**I**) fumonisin B_1_. The solid black, red and blue lines indicated fitting of single exponential functions. Experimental conditions: transmembrane potential of 40 mV, solution (4 M KCl, Tris-HCl 5 mM, pH 7.5). Mycotoxin concentrations: 3.5 µM (OTA, AFB_1_ and FB_1_).

Voltage-clamp conditions were used to investigate the interaction of individual mycotoxins molecules with a single alphatoxin nanopore. In a 4 M KCl solution (pH 7.5) and a transmembrane potential of 40 mV, the unitary alphatoxin nanopore presents a constant current of approximately 150 pA and 4 nS conductance; these parameter values are in agreement with those reported in several previous studies [42,48,52,53]. Adding ochratoxin A, aflatoxin B_1_ or fumonisin B_1_ to the KCl solution in the trans side of unitary alphatoxin nanopore resulted in reversible and characteristic blockage events of the ionic current. A representative single nanopore ionic current profiles for interaction of the alphatoxin nanopore with the ochratoxin A, aflatoxin B_1_ and fumonisin B_1_ are shown in Figure 1A,D,G, respectively. Note that blockage events are indicated by dashed black rectangles. Figure 1B,E,H shows the blockage events can be observed as fluctuation discrete in the ionic current. This behavior is similar to that reported in studies of other analytes, indicating that this nanopore can be used in mycotoxin detection [48,49]. Figure 1C,F,I depicts the respective histograms of dwell times for the ochratoxin A, aflatoxin B_1_ and fumonisin B_1._ Mean dwell time values (τ_off_) and mean interevent interval values (τ_on_) were obtained from the dwell histograms, and the interevent histograms were fitted to single exponential functions [54,55].

**Figure 2:**
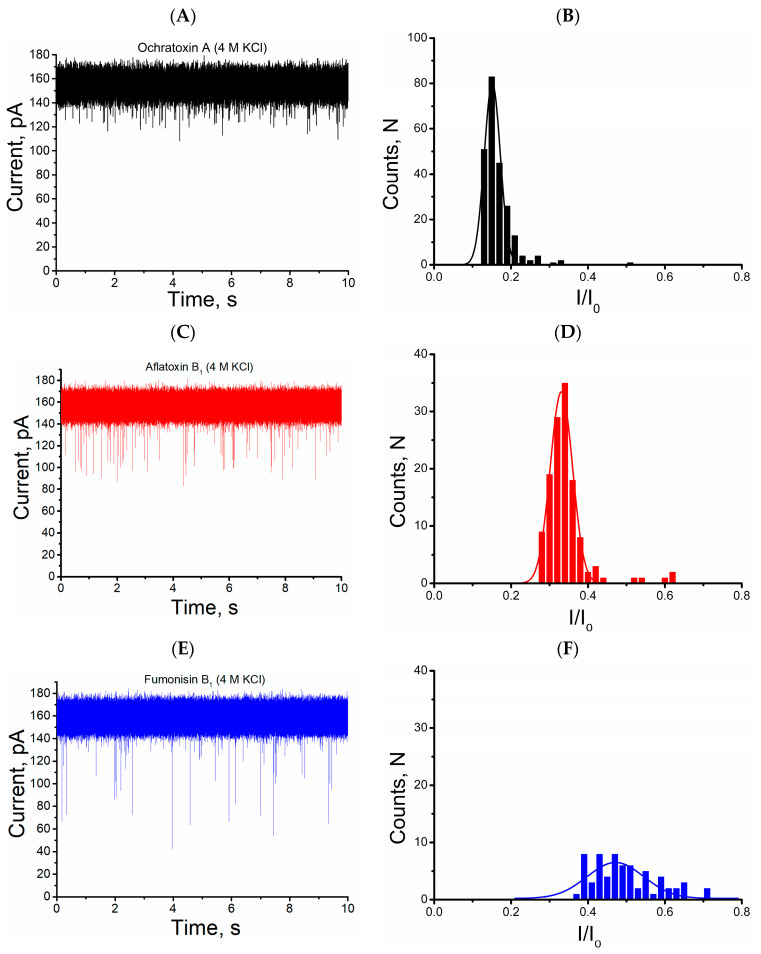
Single alphatoxin nanopore detection of mycotoxins. Representative single nanopore ionic current trace for interaction of the alphatoxin nanopore with mycotoxin (**A**) ochratoxin A, (**C**) aflatoxin B_1_ and (**E**) fumonisin B_1_ (**D**). Histograms of normalized blockage current (I/I_0_) of mycotoxins (**B**) ochratoxin A, (**D**) aflatoxin B_1_ and (**F**) fumonisin B_1_. The solid black, red, and blue lines are Gaussian fit to the histograms of ochratoxin A, aflatoxin B_1_ and fumonisin B_1_, respectively. Experimental conditions: transmembrane potential of 40 mV, solution (4 M KCl, Tris-HCl 5 mM, pH 7.5), Mycotoxin concentrations: 3.5 µM OTA, 10 µM AFB_1_ and 3.5 µM FB_1_.

Blockage events were characterized based on the mean dwell time (τ_off_) and residual current ratio (I/I_0_; I denotes the magnitude of current decrease, and I_0_ denotes open nanopore current) [54,55]. Analysis of ionic current tracings reveal the presence of three I/I_0_ and τ_off_ values for each of the mycotoxins: ochratoxin A, I/I_0_ = 0.15 ± 0.001, 0.0311 ± 0.006 ms (*n* = 3), respectively; aflatoxin B_1_, I/I_0_ = 0.326 ± 0.025, 0.059 ± 0.007 ms (*n* = 3), respectively; and fumonisin B_1_, I/I_0_ = 0.443 ± 0.025, 0.0996 ± 0.011 ms (*n* = 3), respectively (Figure 1 and Figure 2).

The capacity of the single alphatoxin nanopore can be used in directly detecting these mycotoxins, reinforcing the potential to use this nanopore for analyzing small molecules, as demonstrated in previous studies [43,44,45,46,47].

**Figure 3:**
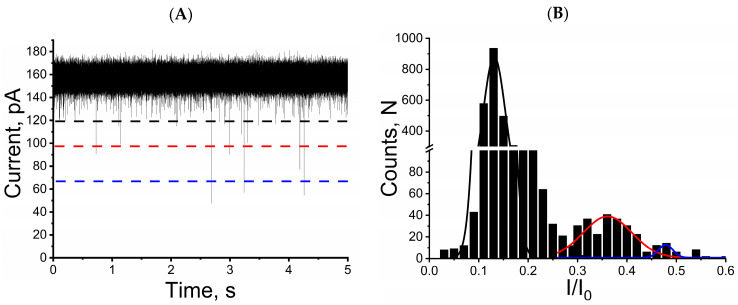
Unitary alphatoxin nanopore detection can differentiate between mycotoxins in aqueous solution. (**A**) Representative single nanopore ionic current trace of mycotoxins in mixture interacting with alphatoxin nanopore. The horizontal dashed black, red and blue lines indicate the blockage amplitudes of ochratoxin A, aflatoxin B_1_ and fumonisin B_1_, respectively. (**B**) Histograms of normalized blockage current (I/I_0_) of mycotoxins mix (ochratoxin A, aflatoxin B_1_ and fumonisin B_1_). The solid black, red and blue lines are Gaussian fit to the histograms of ochratoxin A, aflatoxin B_1_ and fumonisin B_1_, respectively. Experimental conditions: transmembrane potential of 40 mV, solution (4 M KCl, Tris-HCl 5 mM, pH 7.5). Mycotoxin concentrations: 10 µM OTA or AFB_1_ and 3.5 µM FB_1_.

Furthermore, simultaneous addition of three mycotoxins to the solution in the trans side of the unitary alphatoxin nanopore (at concentrations of 10 µM for OTA or AFB_1_ and 3.5 µM for FB_1_) induced three distinct patterns of ionic current blockage (Figure 3A). The amplitude of these three blockage profiles is similar, as can be seen in Figure 1 and Figure 2. Therefore, the single alphatoxin nanopore facilitates differentiating between these mycotoxins when simultaneously present in aqueous solution. The discrimination process is based on the calculation of the residual current ratio and the residence time of each mycotoxin in the unitary alphatoxin nanopore (Figure 3B).

Analysis of the ionic current tracings obtained from the single alphatoxin nanopore in the simultaneous presence of the three mycotoxins resulted in the following (I/I_0_) and τ_off_ values: 0.145 ± 0.021, 0.028 ± 0.004 ms (*n* = 3) for OTA; 0.364 ± 0.005, 0.061 ± 0.007 ms (*n* = 3) for AFB_1_; and 0.476 ± 002, 0.095 ± 0.006 ms (*n* = 3) for FB_1_. These values are in accordance with the results obtained for the single alphatoxin nanopore in the presence of only one type of mycotoxin (Figure 1 and Figure 2).

These results, together with recent studies demonstrating that unitary alphatoxin nanopore detection can be used to discriminate between microcystins, reinforce the concept of using this nanopore as a potential molecular tool for the discrimination of mycotoxins in aqueous solution [48].

**Figure 4:**
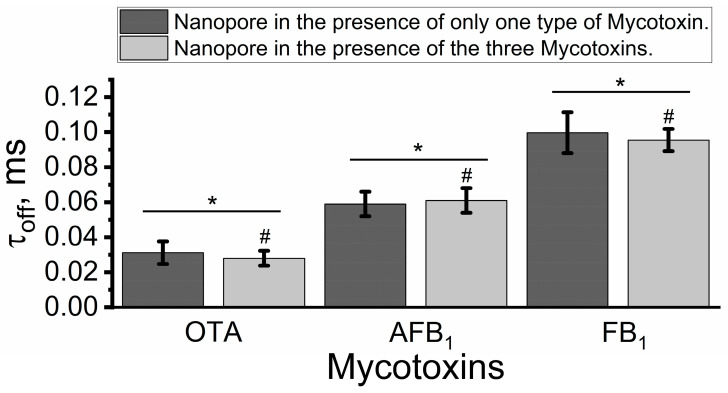
Residence time is a discriminant for multivariate analysis of mycotoxins in aqueous solution. Experimental conditions: transmembrane potential of 40 mV, solution (4 M KCl, Tris-HCl 5 mM, pH 7.5), and 3.5 µM OTA, AFB_1_ and FB_1_, alone or in mixture. Values represent mean ± S.D. of three independent experiments. # Indicates that residence time in the nanopore is not statistically different when in the presence of only one type of mycotoxin. Student’s *t*-test, *p* > 0.05. * Data are statistically different for each type of mycotoxin. ANOVA, *p* < 0.05.

Figure 4 demonstrates the residence times of mycotoxins in the single alphatoxin nanopore. There were no differences between the values of the mycotoxins’ residence times in the nanopore when in the presence of only one type of mycotoxin or in the presence of a mixture of mycotoxins (Figure 4). Therefore, the unique residence time for each type of mycotoxin in the unitary alphatoxin nanopore allows for their identification and serves as a second discriminant for multivariate analysis of these toxin classes in aqueous media [48,56,57].

### 2.2. Augmentation of the Salt Concentration Increases the Frequency of Ionic Current Blockage in the Unitary Alphatoxin Nanopore

Recent studies have updated methodologies to improve the electrophysiological measurements generated from biosensors that use synthetic or biological nanopores. Changes in the saline concentration or the making of concentration gradients on the experimental solution are ways to improve the sensitivity of this biosensors [50,51,52,53,54,55]. Among these studies, some used the first approach, demonstrating that the detection capacity and sensitivity of the nanopore can be strongly influenced not only by the composition of the ionic solution, but also by the action of solutions with high ionic concentration [48,53].

**Figure 5:**
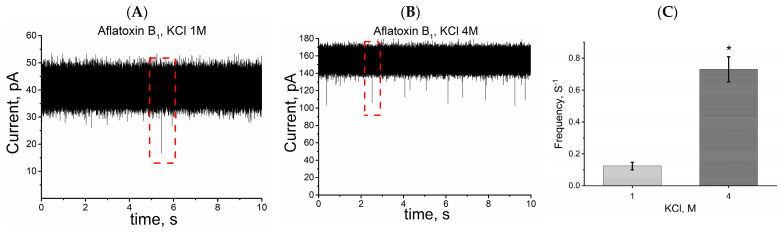
Higher KCl concentration increases the frequency of blockage events induced by mycotoxins. Representative single nanopore ionic current trace of AFB_1_ interaction with the alphatoxin nanopore in KCl at (**A**) 1 M and (**B**) 4 M. (**C**) Increased frequency of blockage events caused by KCl concentration. Experimental conditions: transmembrane potential of 40 mV, 1 M or 4 M KCl and 5 mM Tris, pH 7.5, and 3.5 µM AFB_1_. Red dashed rectangles indicate blockage events. * Data are statistically different from results for 1 M KCl. Student’s *t*-test, *p* < 0.05.

In this case, Figure 5 illustrates typical single nanopore ionic current traces of the aflatoxin B_1_ interaction with alphatoxin nanopore; with larger blockage frequency values for 4 M KCl solution, indicating that increasing the potassium chloride concentration from 1 to 4 M increases the frequency of ionic current blockage events by approximately five times. Previous data and the results of this work clearly demonstrate that increasing the salt concentration enhances the capability of stochastic detection using protein pores, such as the alphatoxin nanopore [53,58]. Other studies have demonstrated that an increasing salt gradient favorably influences the capture of molecules with residual charge (DNA, for example) via the protein or synthetic nanopore, but only if the analyte is placed where the salt concentration is lowest. This behavior is due to electrostatic focusing [50,51]. Our results were obtained from independent experiments carried out with symmetric KCl solutions, that is, at concentrations of 1 M or 4 M of KCl on both sides of the single alphatoxin nanopore, therefore, we do not have the presence of a saline gradient. Thus, the increase in the frequency of ionic current blocking events denoted here is probably due to the salting-out effect, as already reported in previous studies [42,52,53,57].

### 2.3. Sensitivity of the Unitary Alphatoxin Nanopore to Mycotoxins

**Figure 6:**
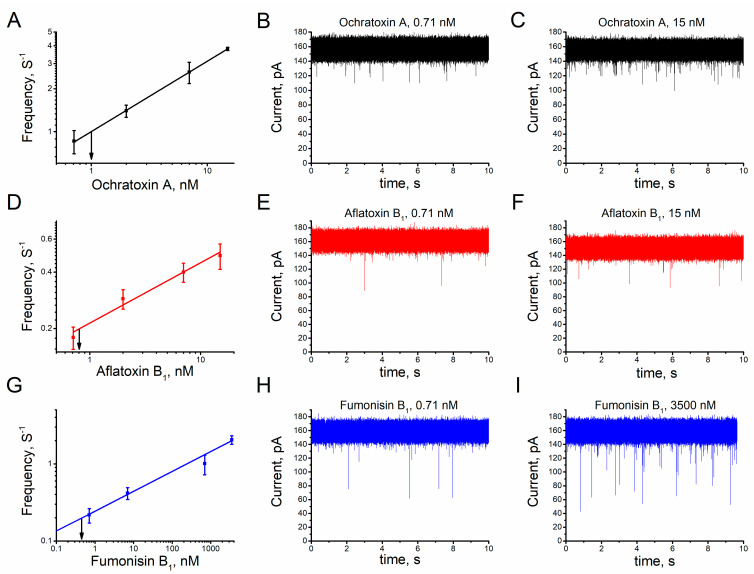
The relationship between frequency of blockage events and mycotoxin concentrations. Calibration curves of the unitary alphatoxin nanopore to mycotoxins: (**A**) vertical arrows indicate the background equivalent ochratoxin A concentration at an event frequency of 1 Hz; and (**D**,**G**) vertical arrows indicate the background equivalent aflatoxin B_1_ or fumonisin B_1_ concentration at an event frequency of 0.2 Hz. Representative single nanopore ionic current interaction of alphatoxin nanopore with mycotoxins (**B**) ochratoxin A, 0.71 nM; (**E**) aflatoxin B_1_, 0.71 nM; (**H**) fumonisin B_1_, 0.71 nM; (**C**) ochratoxin A, 15 nM; (**F**) aflatoxin B_1_, 15 nM; and (**I**) fumonisin B_1_, 3500 nM. Experimental conditions: transmembrane potential of 40 mV, solution (4 M KCl, 5 mM Tris−HCl, pH 7.5).

The search for alternative and promising tools for mycotoxin detection results in the expansion of studies in the field of analysis [29,30,31,32,33,34]. Studies denote the emergence and advancement of methodologies based on biosensors (mainly immunosensors) used to detect this class of toxins [59,60]. Application of the single alphatoxin nanopore as a recognition element has shown satisfactory results in the stochastic sensing of toxins in aqueous solution [48]. Their properties of high sensitivity, low detection limit values and high dynamic range values indicate that sensors based on the unitary alphatoxin nanopore have beneficial characteristics for the detection of single molecules [35,37,42,61]. Gold-standard techniques are constantly being updated with dynamic range in the nano to picomolar order [28]. The amplitudes between the highest and lowest concentrations in which analytes can be discriminated represents the dynamic range of a sensor [62,63]. A recent study used mycotoxin-specific aptamers to demonstrate use of the single alphatoxin nanopore in detecting ochratoxin A at levels recommended by control agencies [49]. In this study, the increase in KCl concentration allowed use of the single alphatoxin nanopore in discriminating and detecting the studied mycotoxins at the nanomolar level. The dynamic range was estimated at approximately 0.71 to 15 nM for ochratoxin A and aflatoxin B_1_ and, 0.71 to 3500 nM for fumonisin B_1_ (Figure 6 and Figure S1).

The slope value of the calibration curve of a sensor indicates its sensitivity [62]. Therefore, the slope of the calibration curve was calculated to determine the sensitivity of the single alphatoxin nanopore for each mycotoxin. The single alphatoxin nanopore sensitivity values (nM s^−1^) are 0.148 ± 0.001 (ochratoxin A), 0.09 ± 0.010 (aflatoxin B_1_) and 0.257 ± 0.016 (fumonisin B_1_) (Figure 6). The frequency of blockage events increases linearly with the mycotoxin concentration (Figure 6). Therefore, it was possible to determine the concentration of mycotoxins in solution using the unitary alphatoxin nanopore; the dependence between the frequency of blockage events and the mycotoxin concentration was used to calculate the detection limit. The detection limit is the lowest analyte concentration value detectable by the single alphatoxin nanopore for a certain frequency value of ionic current blocking events [52,53]. Figure 6 shows the ability of the nanopore to detect mycotoxins in aqueous solution (assessment at practical 0.2 Hz of events), estimated at 0.8 nM for aflatoxin B_1_ (Figure 6D) and 0.45 nM for fumonisin B_1_ (Figure 6G). Likewise, adopting a 1 Hz frequency, it was possible to estimate the limit detection of the ochratoxin A as approximately 1.0 nM (Figure 6A). This value is within the 0.3 to 16 nM range of the maximum allowable levels for this class of toxins in food [59].

It is important that strategies are underway for the development of portable instrumentation [49] and microfluidic systems [44] that will allow the use of detection systems to advance based on the applicability of biosensors. Given the success of the alphatoxin nanopore biosensor, it is important to expand the technique for detecting new compounds and evaluate parameters that improve the stability and sensitivity of these analysis [44,48,49,50,53,54]. Previous studies show the usefulness of the alphatoxin nanopore biosensor in combination with aptamers in the analysis of ochratoxin A from corn samples [49]. The sensitivity values of the technique were in the range of 1.69 pM under the experimental conditions proposed in the study, allowing the analysis of real samples with concentrations starting from 0.2 nM OTA [49]. The use of aptamers combined to the alphatoxin nanopore has also been used for the detection of other chemical substances, such as cocaine [44]. However, this type of analysis is limited to the specificity of the aptamer and also makes the technique more laborious [44,49]. Other new techniques based on the applicability of chemiluminescence immunosensors and competitive magnetic immunodetection provide quite satisfactory detection limits for AFB_1_ in the order of picomolar [34,60]. Therefore, it is necessary to find alternatives to the use of aptamers and antibodies, since the use of these techniques for mycotoxins analysis requires more steps and costs. We present here the perspective of the alphatoxin biosensor for the detection and discrimination of mycotoxins representing the three main groups of fungi (AFB_1_, OTA, and FB_1_). The values of 0.8 nM (AFB_1_), 0.45 nM (FB_1_) and 1 nM (OTA) obtained in this study are lower than those recommended by legislation for different foods [59]. Studies carried out for the detection of other toxins, such as microcystins, have already demonstrated the capacity of the biosensor for multianalytical detection in the absence of molecular adapters [48]. This shows that the alphatoxin nanopore biosensor is able to be used in the future for the analysis of samples extracted from food.

In addition, the method presented in this study considerably simplifies assay procedures and reduces analysis time compared with standard methods, as it is not necessary to mark, modify or conjugate the mycotoxins with other types of molecules (aptamers or antibodies, for example) or nanoparticles. Additionally, the capacity of unitary alphatoxin nanopore to detect mycotoxins can be improved using several strategies, such as changing the ionic composition of the lipid membrane bathing solution and application of a saline gradient.

## 3. Conclusions

In this study, we demonstrate that increasing the KCl concentration in solution causes an increase in the frequency of blockage of the ionic current induced by the presence of mycotoxins in aqueous lumen of the single alphatoxin nanopore. Consequently, the stochastic sensing capacity of unitary alphatoxin nanopore is improved, enabling the discrimination of three relevant agricultural mycotoxins: ochratoxin A, aflatoxin B_1_ and fumonisin B_1_. In addition, the sensitivity is increased, allowing a single alphatoxin nanopore to be used in the detection of these mycotoxins at the nanomolar level, which is comparable to the standard techniques used for quantification of these molecules. Finally, its ability to detect and discriminate mycotoxins at a nanomolar level indicates that the unitary alphatoxin nanopore is a promising molecular tool for discriminatory analysis of mycotoxins in aqueous solution.

## 4. Materials and Methods

### 4.1. Materials and Chemicals

Wild type alphatoxin from *Staphylococcus aureus* was purchased from Calbiochem (Madison, WI, USA); 1,2-diphytanoyl-sn-glycero-3-phosphocholine (DPhPC) was acquired from Avanti Polar Lipids (Birmingham, AL, USA); and 2-amino-2-hydroxymethyl-1,3-propanediol (Tris) and citric acid were acquired from Fluka (Buchs, Switzerland). High quality (>99.99%) potassium chloride, n-hexane, and solution analytical standards of aflatoxin B_1_, ochratoxin A and fumonisin B_1_ were acquired from Merck (Darmstadt, Germany). High-purity water was prepared using a Milli-Q Plus purification system (Billerica, MA, USA). All chemicals and solvents were of analytical grade and used as received.

### 4.2. Planar Lipid Bilayer Formation, Insertion of Single Alphatoxin Nanopore and Data Analysis

All experiments to obtain the solvent-free planar bilayer lipid membranes with a capacitance of 40 pF were performed using the lipid monolayer apposition technique and DPhPC in hexane at 25 ± 1 °C, as previously described [52,64]. The lipid bilayers were mounted in the orifice (diameter of approximately 50 μm) a Teflon film serving as a divider for two Teflon hemi-chambers. The membrane bathing solution was composed of 1 M KCl or 4 M KCl and 5 mM Tris−HCl buffered to pH 7.5. We chose to use mainly high concentrations of KCl in each Teflon hemi-chamber, since previous studies have demonstrated improvements in the detection capacity of the single alphatoxin nanopore [48,53]. If not mentioned specially, the applied transmembrane potential was 40 mV. After membrane formation, approximately 0.5 µL of alphatoxin stock solution (0.07 mg/mL) was added to the bath solution on the cis side of the experimental hemi-chamber for a final concentration of approximately 2 ng/mL, an amount sufficient to promote the incorporation of a single alphatoxin nanopore. The Ochratoxin A, Aflatoxin B_1_, and Fumonisin B_1_ were added to the trans side of the hemi-chamber, from stock solutions at concentrations ranging from 1 mM to 1 nM.

An Axopatch 200B amplifier (Molecular Devices, San Jose, CA, USA) in voltage-clamp mode was used to measure the current travelling through the lipid bilayer membrane. Transmembrane potential was sustained using Ag/AgCl electrodes in 3 M KCl 2% agarose bridges montaged in standard 200 mL pipette tips. Currents were acquired using an Axopatch 200B amplifier with a Bessel filter at 10 kHz and sampled at 50 kHz using an IBM computer connected to Digidata 1440 A (Molecular Devices, San Jose, CA USA).

Analysis of the single nanopore ionic current traces and determination of the sojourn time, amplitude and frequency of blockage events and kinetic constants of mycotoxin–nanopore interactions were performed as reported in previous studies [45,46]. Briefly, the characteristic time, τ_on_, was obtained from the collected time intervals between the end of one blockage event and the onset of the next. The residence time, τ_off_, was obtained from the collected duration time of blockage events. Finally, Clampfit 10.7 (Molecular Devices, San Jose, CA, USA) was used to analyze single nanopore ionic current trace, and Origin 8.1 (Origin, Version 8.1. OriginLab Corporation, Northampton, MA, USA) was used to generate histograms and for plotting and curve fitting.

## Data Availability

Not applicable.

## Supplementary Materials

The following supporting information can be downloaded at: https://www.mdpi.com/article/10.3390/toxins15030183/s1, Figure S1. Calibration curves of the unitary alphatoxin nanopore and current traces (OTA and AFB_1_).
