# Supplementary material for: Alphatoxin Nanopore Detection of Aflatoxin, Ochratoxin and Fumonisin in Aqueous Solution
Source: Toxins (Basel). 2023 Feb 28;15(3):183. doi: 10.3390/toxins15030183 (PMC10058818; doi:10.3390/toxins15030183)
Supplement: Supplementary file 1 [file toxins-15-00183-s001.zip › toxins-2203955-supplementary.pdf]

toxins

Supplementary Materials: Alphatoxin Nanopore Detection of Aflatoxin, Ochratoxin and Fumonisin in Aqueous Solution

Artur Alves Rodrigues da Silva, Janilson José da Silva Júnior, Maria Isabel dos Santos Cavalcanti, Dijanah Cota Machado, Paloma Lys Medeiros and Claudio Gabriel Rodrigues

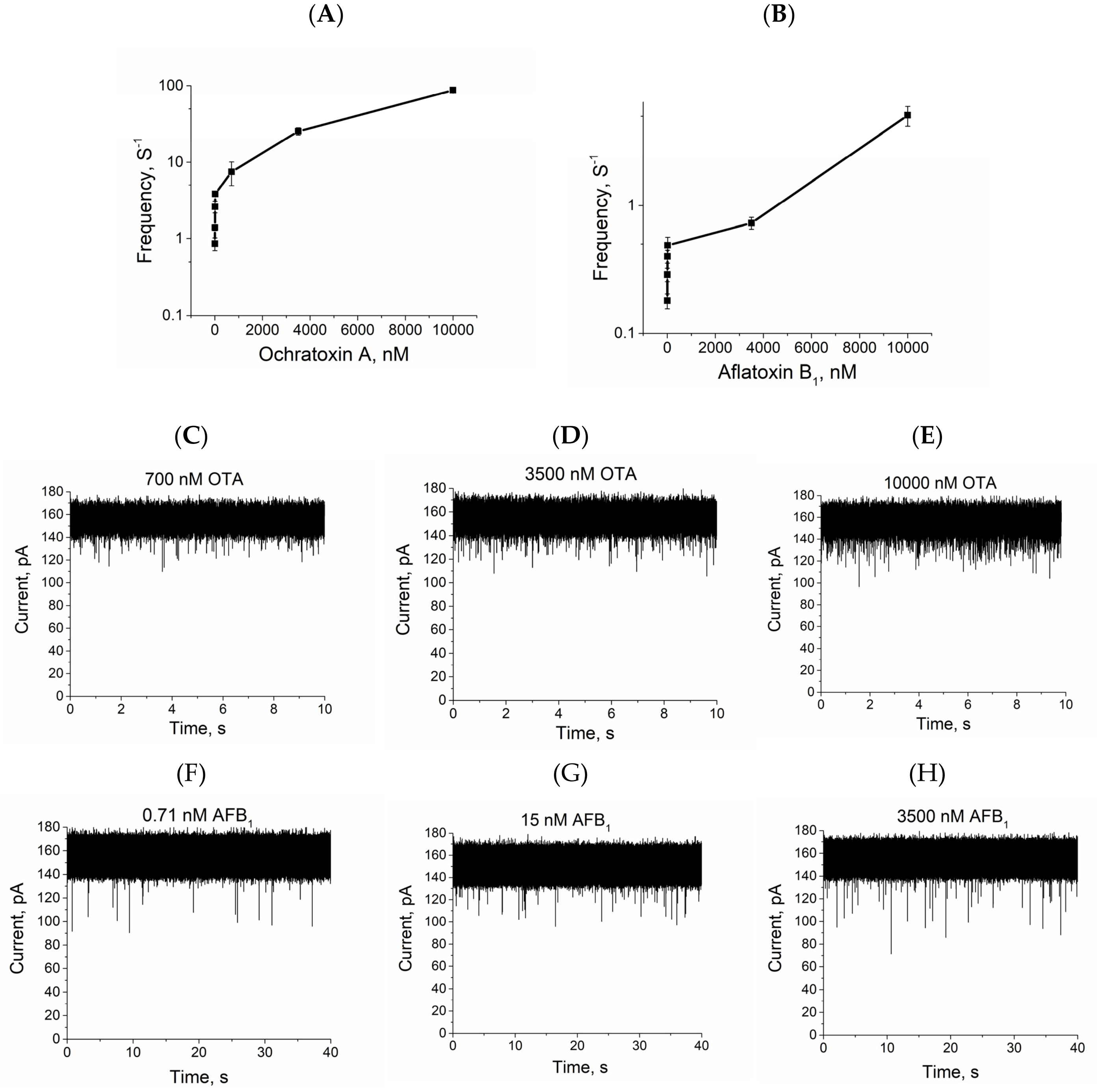

Figure S1. Calibration curves of the unitary alphatoxin nanopore and current traces (OTA and AFB_1). Calibration curve equivalent ochratoxin A **(A)** and aflatoxin B_1 **(B)** concentration in ratio event frequency. Alphatoxin nanopore current traces of OTA in high concentrations in calibration curve **(C-E)**. Alphatoxin nanopore current traces of AFB_1 at different concentrations in the calibration curve, the analysis time was increased to better show the variation of concentration in ratio event frequency **(F-H)**.
